# Cognition, learning behaviour and hippocampal synaptic plasticity are not disrupted in mice over-expressing the cholesterol transporter ABCG1

**DOI:** 10.1186/1476-511X-8-5

**Published:** 2009-02-24

**Authors:** Pamela F Parkinson, Timal S Kannangara, Brennan D Eadie, Braydon L Burgess, Cheryl L Wellington, Brian R Christie

**Affiliations:** 1 The Department of Pathology and Laboratory Medicine, Child and Family Research Institute, University of British Columbia, Vancouver, BC, Canada; 2 Division of Medical Sciences, University of Victoria, Victoria, BC, Canada; 3 The Brain Research Centre, University of British Columbia, Vancouver, BC, Canada; 4 The Graduate Program in Neuroscience, University of British Columbia, Vancouver, BC, Canada; 5 The Department of Cellular and Physiological Sciences, University of British Columbia, Vancouver, BC, Canada; 6 MD/PhD program, University of British Columbia, Vancouver, BC, Canada; 7 The Department of Biology, University of Victoria, Victoria, BC, Canada

## Abstract

**Background:**

Cognitive deficits are a hallmark feature of both Down Syndrome (DS) and Alzheimer's Disease (AD). Extra copies of the genes on chromosome 21 may also play an important role in the accelerated onset of AD in DS individuals. Growing evidence suggests an important function for cholesterol in the pathogenesis of AD, particularly in APP metabolism and production of Aβ peptides. The ATP-Binding Cassette-G1 (ABCG1) transporter is located on chromosome 21, and participates in the maintenance of tissue cholesterol homeostasis.

**Results:**

To assess the role of ABCG1 in DS-related cognition, we evaluated the cognitive performance of mice selectively over-expressing the ABCG1 gene from its endogenous regulatory signals. Both wild-type and ABCG1 transgenic mice performed equivalently on several behavioral tests, including measures of anxiety, as well as on reference and working memory tasks. No deficits in hippocampal CA1 synaptic plasticity as determined with electrophysiological studies were apparent in mice over-expressing ABCG1.

**Conclusion:**

These findings indicate that although ABCG1 may play a role in maintaining cellular or tissue cholesterol homeostasis, it is unlikely that excess ABCG1 expression contributes to the cognitive deficits in DS individuals.

## Background

The central nervous system is the most sterol-rich organ in the body, containing over 25% of total body cholesterol in only 2% of total body weight [1]. Cholesterol cannot cross the blood brain barrier; therefore, sterol homeostasis in the brain is largely regulated independently from that in peripheral tissues. Aberrant brain cholesterol metabolism has been associated with compromised Aβ metabolism, cognitive function, and synaptogenesis [2]. In addition, a link between brain cholesterol metabolism and learning and memory was recently observed with the demonstration that mice deficient in 24-hydroxylase do not show long-term potentiation (LTP) and are profoundly impaired in spatial learning tasks [3]. Understanding how genes that regulate sterol homeostasis in the brain affect cognitive and neuronal function may therefore lead to insights into several disorders of the central nervous system.

ABCG1 is the founding member of the ABCG subclass of the ATP-Binding-Cassette (ABC) transporter superfamily [4]. Like several other members of the ABCG family, ABCG1 functions in the regulation of sterol homeostasis and high density lipoprotein (HDL) metabolism. ABCG1 is widely expressed in a variety of tissues, with the highest levels of expression observed in macrophage-rich tissues such as the spleen, lungs, and thymus; correspondingly, the majority of studies concerning ABCG1 function focused on the peripheral tissues. There are, however, several lines of evidence supporting the idea that ABCG1 may have an important role in the CNS, particularly concerning neurodegenerative disorders. ABCG1 is abundantly expressed in the brain, including the hippocampal formation [5,6]. Additionally, ABCG1 mRNA and protein levels are significantly increased in DS cortex, and over-expression of ABCG1 affects Aβ production in transfected cells [7]. Interestingly, selective over-expression of ABCG1 *in vivo *did not affect Aβ or amyloid levels in the PDAPP mouse model of AD (Burgess et al., 2008), suggesting that over-expression of ABCG1 is unlikely to contribute to the early onset of AD in the DS population. As ABCG1 *does *alter cholesterol homeostasis in the brain (Burgess et al., 2008), it therefore remains possible that excess ABCG1 may influence neuronal physiology and contribute to the cognitive deficits in DS individuals. The purpose of the present study was to investigate the role of ABCG1 on cognition, learning behaviour and synaptic plasticity in the hippocampus.

## Methods

### Mice

All procedures were approved by the University of British Columbia Animal Care Committee and are in accordance with the Canada Council on Animal Care. ABCG1 bacterial artificial chromosome (BAC) transgenic mice were generated as previously described [6]. All animals were group housed in standard cages in a colony maintained at 21°C. Animals were maintained on a 12-hour light/dark cycle with access to food and water *ad libitum*.

### ABCG1 Protein Expression Assay

To investigate protein expression of ABCG1 in adult brain, mice were anesthetized by intraperitoneal administration of 600 mg/kg Avertin (Sigma-Aldrich) or intramuscular administration of a 20 mg/kg xylazine (Bayer) and 150 mg/kg ketamine (Bimeda-MTC) mixture, then perfused for 7 min with phosphate buffered saline (PBS) containing 2500 U/L Heparin. Brains were collected and dissected into cortex, hippocampus, and cerebellum, and snap frozen at -80°C until analysis. ABCG1 protein expression was detected in a crude total membranes preparation as described [8]. Briefly, tissues were homogenized in 5 volumes of lysis buffer (50 mM mannitol, 2 mM EDTA, 50 mM Tris HCl pH 7.6, and Complete protease inhibitor (Roche)), and centrifuged at 500 × g to pellet nuclei and debris. Between 400–450 μl of supernatant was layered onto 600 μl of fractionation buffer (300 mM mannitol, 2 mM EDTA, 50 mM Tris HCl pH 7.6) and centrifuged at 100,000 × g for 45 min to pellet total membranes. Membranes were resuspended in 150–200 μl of lysis buffer. SDS was added to a final concentration of 1% prior to Tris-glycine SDS-PAGE and immunoblotted with antibodies against ABCG1 (Novus) and Na/K-ATPase (Novus) as an internal loading control.

### Behavioural Analysis

Behavioural analysis cohorts included a total of 22 female mice, with 11 ABCG1 BAC Tg mice and 11 wild-type littermate controls. Phenotypic assessment of ABCG1 BAC Tg mice was carried out using the SHIRPA protocol [9] to screen for any differences in baseline phenotype, as these could markedly affect performance on cognitive tests.

Locomotor activity, exploratory behaviour and general anxiety were analyzed using the open field test. A circular arena with a diameter of 90 cm was set up directly underneath an overhead digital camera (Logitech QuickCam Pro 5000) in a brightly lit room in an undisturbed, quiet location. A room divider shielded the experimenter and computer from the animal's view for the duration of the task. Animals were brought into the testing room, and given 5–10 minutes to adjust to the room's environment in their home cages. Each mouse was placed in the arena individually, and allowed to freely explore for 5 minutes, while its activities were tracked and recorded using ANY-maze™ (Stoelting Co.). Upon completing the task, the mouse was removed from the arena by the experimenter and returned to the home cage. The number of defecations in the arena was recorded for each mouse, and the arena was cleaned with a mild soap following each test to avoid transfer of olfactory cues between animals. Distance traveled, average speed and path length serve as measures of locomotor activity and exploratory behaviour. Anxiety was assessed through measurement of time spent around the edges of the arena relative to the time spent in the centre, and also by freezing and defecation.

Emotionality was specifically measured using the elevated plus maze: an elevated maze with 2 open arms and 2 enclosed arms, set up in a room in an undisturbed, quiet location. The experimenter and computer were behind a room divider, hidden from the animal's view for the duration of the task. Animals were given 5–10 minutes in their home cages to adjust to the testing room. Each mouse was initially placed in the centre of the maze, and allowed 5 minutes of free exploration. The animal's activities were tracked and recorded by an overhead camera (Logitech QuickCam Pro 5000) and ANY-maze™. Upon completion of the task, the mouse was returned to its home cage and number of defecations was recorded before the maze was cleaned with a mild soap prior to the next trial.

### Assessment of Learning and Memory

Hippocampal-dependent learning and memory was assessed using several variations of the Morris Water Maze task. The water maze consisted of a circular white pool (100 cm diameter, 54 cm height) filled with water to a height of 36 cm. The water was rendered opaque using white, non-toxic tempera paint. A circular platform (14 cm diameter, 35 cm height) was submerged in the pool (about 1 cm below the surface), providing a ledge upon which the mice could step to escape from the water. For each trial, a mouse was released into the pool at one of three release positions and allowed to search for the platform. Each trial lasted a maximum of 60 seconds, after which the mouse was manually guided to the platform and allowed to remain on the platform for another 10 seconds. All trials were tracked using an overhead digital camera (Logitech QuickCam Pro 5000) and ANY-maze™ computer software.

The visible platform task was used to screen for any visual deficits, as well as motor problems (such as inability to swim) that may hinder an animal's ability to complete water maze training trials. The platform was located about 2 cm above the surface of the water, and a visually conspicuous marker, such as a small coloured flag, was attached to the top of the platform. The platform location was changed for each of the 4 trials. Mice were released into the pool and allowed 60 seconds to locate the platform, while being tracked and recorded by ANY-maze™. The experimenter noted any apparent deficits in vision or motor behaviors.

The reference memory task was used to assess learning and long-term memory for a fixed platform location. Distinct geometric shapes were attached to the walls of the pool on three sides, and the experimenter and computer system were hidden behind a dividing wall throughout the experiment to avoid influence of extraneous cues. Mice were trained for 4 trials per day, for a total of 5 days. Twenty-four hours after the last training session, a probe trial was administered, in which the platform was removed from the pool and the mouse was allowed to swim freely for the full 60 seconds. Time spent in the quadrant of the pool that had previously contained the platform was measured as an indication of memory retention.

### Electrophysiology

For *in vitro *electrophysiology, aged animals were anesthetized with isofluroane and decapitated. The brain was removed and immersed in ice-cold sucrose artificial cerebrospinal fluid (sACSF; pH7.2) containing (in mM): 110.00 sucrose, 60.00 NaCl, 3.00 KCl, 1.25 NaH_2_PO_4_, 28.00 NaHCO_3_, 0.50 CaCl_2_, 7.00 MgCl_2_, 5.00 dextrose and 0.60 ascorbate, oxygenated with 95%O_2_/5%CO_2_. Transverse hippocampal slices (350 μm) were generated using a Vibratome Sectioning System 1500 (Ted Pella, Redding, CA). Slices were gently transferred to an incubation chamber filled with oxygenated normal artificial cerebrospinal fluid (ACSF; pH 7.2) containing (in mM): 125.0 NaCl, 2.5 KCl, 1.25 NaH_2_PO_4_, 25.0 NaHCO_3_, 2 CaCl_2_, 1.3 MgCl_2_, and 10 dextrose, and maintained at 30°C for a minimum of 1 hour post-dissection. Slices were then transferred to a recording chamber superfused at a rate of 2 ml/min with 30°C, oxygenated ACSF. A recording electrode (0.7–1.5 MΩ) filled with ACSF was placed under visual guidance into the striatum radiatum of the hippocampal CA1 region using an Olympus BX51W1 microscope. Field excitatory postsynaptic potentials (fEPSPs) were evoked using monophasic negative current pulses (120 μs, 10–80 μA) supplied to the CA1 Schaeffer Collaterals via a concentric bipolar stimulating electrode (FHC, Bowdoin, ME) connected to a digital stimulus isolation unit (Getting Instruments, San Diego, CA). Responses were acquired at 100 Hz using a MultiClamp 700B amplifier (Molecular Devices, Sunnyvale, CA) and collected on a computer for offline analysis. For each slice, stimulus intensity was adjusted to yield 50–55% of the maximal response. Prior to baseline measurements, a paired-pulse protocol (50 ms inter-stimulus interval) was employed. Baseline measurements were collected using individual fEPSPs evoked every 15 seconds. A steady baseline of 15 minutes was required for all responses. Following baseline acquisition, a conditioning protocol was applied: high frequency stimulation (HFS: four bursts of 50 pulses at 100 Hz, 30 s between bursts) to induce long-term potentiation. Immediately after the conditioning protocol, baseline measurements were acquired for a minimum of 1 hour. Following the hour of post acquisition, a second paired-pulse protocol (50 ms inter-stimulus interval) was conducted. Computed results were processed for statistical analysis using Clampfit (Molecular Devices, Sunnyvale, CA), Excel 2007 (Microsoft) and Statistica 7.0 (Statsoft). For all studies, data was presented as means ± standard error of the mean (S.E.M.) and compared using unpaired *t*-tests. Differences were considered significant when *P *< 0.05.

## Results

### ABCG1 Expression in Brains of Transgenic Mice

**Figure 1:**
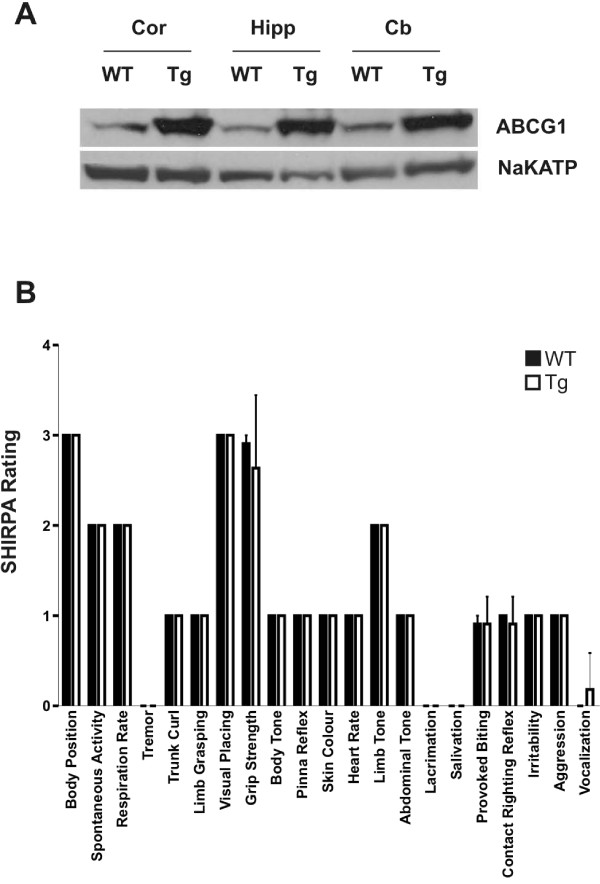
**ABCG1 BAC Tg mice over-express the ABCG1 transporter in the cortex, hippocampus, and cerebellum, and show normal behaviour**. A) Crude membrane fractions were extracted from dissected brain regions and were subjected to Western blot. Blots were probed by polyclonal antibodies recognizing human and murine ABCG1 or Na/K-ATPase as a loading control. ABCG1 protein levels are increased 6-fold in cortex (Cor) and hippocampus (Hipp) and 3-fold in cerebellum (Cer) of ABCG1-BAC-Tg (Tg) mice relative to baseline levels in the equivalent region from wild-type (WT) controls. B) SHRPA primary screen on ABCG1 BAC Tg (ABCG1; n = 11) and wild-type (WT; n = 11) mice. Primary screen involves physiological profiling of each mouse, using a number of test categories and assigning a rating for each mouse in each category. All p values are non-significant when compared to WT animals.

As previously reported [6], over-expression of ABCG1 was observed in the brains of ABCG1 transgenic mice that were generated using a BAC insertion of the full human ABCG1 gene (ABCG1 BAC Tg mice), with protein levels between 3–8 fold higher than that observed in wild-type littermate control animals (Figure 1A). The level of over-expression varied with brain region, with greater over-expression in the cortex and hippocampus as compared to the cerebellum. This verified that our transgene does in fact significantly increase ABCG1 protein levels in the brain over endogenous levels.

### Baseline Behavioural Analysis using SHIRPA

A cohort of 22 female mice (n = 11 ABCG1 BAC Tg, n = 11 wild-type; approximately 10 months of age) were assessed using the categories described by the SHIRPA protocol [9] to test whether over-expression of ABCG1 resulted in any gross phenotypic abnormalities. Observations made using the primary screen of the SHIRPA protocol showed that animals expressing the ABCG1 BAC transgene had a similar baseline phenotype to wild-type littermates (Figure 1B). ABCG1 BAC Tg mice did not display any abnormalities in physiological measures such as heart rate, body tone and grip strength or in behavioural measures such as irritability, aggression and exploratory activity.

### Anxiety and General Locomotor Activity

**Figure 2:**
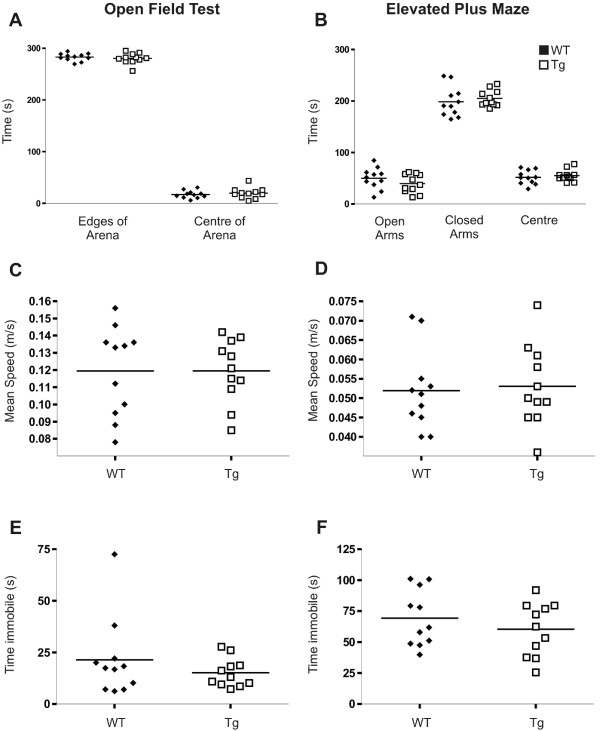
**ABCG1 over-expression does not alter anxiety or general locomotor activity**. ABCG1 BAC Tg (Tg; n = 11) and wild-type mice (WT; n = 11) were assessed for differences in anxiety or locomotor activity using the Open Field Test (A, C, E, G) and the Elevated Plus Maze (B, D, F, H). Data from each test were analyzed to include measures of time spent in maze locations (A, B), mean speed (C, D) and distance (E, F) and time spent immobile (G, H) in the maze. All p values are non-significant.

ABCG1 BAC Tg mice (n = 11) and wild-type littermates (n = 11; approximately 12 months of age) were next evaluated for anxiety level and general locomotor activity, as both factors can interfere with learning tasks and therefore can drastically affect performance during cognitive assessment. The single-trial Open Field Test revealed no difference between groups in time spent in different locations in the arena. Both ABCG1 BAC Tg and wild-type mice spent the majority of their time exploring the edges of the open arena, and generally avoided the brightly lit centre of the arena. No differences were observed when mice were also analyzed for average speed in the arena, distance travelled and time spent immobile or frozen in the arena (Figure 2), indicating that ABCG1 BAC Tg mice have normal exploratory and locomotor behaviour and do not show increased anxiety levels compared to wild-type animals. The Elevated Plus Maze, a behavioural task specifically designed to evaluate anxiety, was administered to verify that anxiety levels are not altered in ABCG1 BAC Tg mice. Wild-type and ABCG1 BAC Tg mice spent comparable amounts of time in the different locations within the maze, with both groups displaying preference for the closed arms of the maze (Figure 2). There were no differences between groups in average speed or distance traveled in the maze. Time spent immobile, an indicator of anxiety, was also similar for both groups.

### Learning and Memory

**Figure 3:**
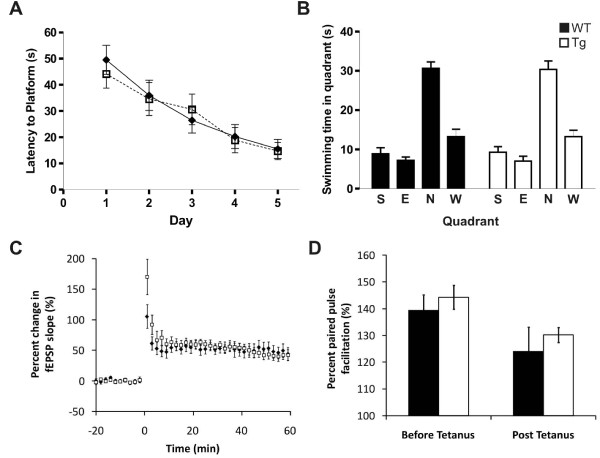
**ABCG1 over-expression does not impact spatial reference memory or synaptic plasticity**. The Morris Water Maze was employed to assess spatial reference memory in wild-type (WT; n = 11) and ABCG1 BAC Tg (Tg; n = 11) animals. (A) Latency to platform. Each point represents an average of 4 daily trials during the training period. No significant difference was observed between the groups in the time taken to find the hidden platform. (B) Probe trial results from a single-trial test. Both control and ABCG1 over-expressing groups showed similar preference for the North (N) quadrant, which contained the platform on training trials. All p values are non-significant. (C) *In vitro *electrophysiology was performed on 350 μm thick hippocampal slices derived from aged mice over-expressing ABCG1 (Tg) and wild-type (WT) littermates. High frequency stimulation (HFS) was applied to the Schaeffer collaterals to induce LTP in the CA1 region. No significant difference was observed between wild-type and transgenic mice. (D) Two stimuli were applied to the Schaeffer collaterals, including paired-pulse facilitation in the CA1 region, before and after the HFS tetanus protocol. No significant differences were observed between wild-type and transgenic mice, indicating that presynaptic release was unaffected by HFS in either mouse phenotype.

Mice expressing the ABCG1 BAC transgene were next evaluated against wild-type littermates on the Morris Water Maze spatial reference memory task. The task consisted of a 5-day acquisition phase in which mice were given 4 trials per day and a maximum time of 60 seconds per trial to locate a platform fixed in the North quadrant. Twenty-four hours later, a probe trial was administered to test recall of the platform location. ABCG1 BAC Tg mice displayed nearly identical average escape latency, or time to locate the platform, over each day of acquisition as compared to wild-type animals, and the learning curve was very similar (Figure 3). These results indicate that ABCG1 over-expression does not significantly affect learning during the acquisition phase of this water maze task. Repeated-measures ANOVA revealed a significant effect of training day, (p < 0.0001), but not group (p = 0.67). Performance on the single-trial probe test was also comparable between groups, with both wild-type and ABCG1 BAC Tg mice spending the greatest amount of time searching in the probe quadrant (North), which previously contained the platform during training days. Both groups displayed strong preference for the North quadrant, indicating that both wild-type and ABCG1 BAC Tg mice were capable of learning the task thoroughly, and were able to remember visual cues associated with the platform location during training trials. Additionally, the comparable performance of ABCG1 BAC Tg and wild-type mice on both learning during the acquisition phase or on memory recall in the probe trial, also suggests that over-expression of ABCG1 alone does not negatively affect hippocampal neuron function involved in the learning and memory required for this task.

### Excess ABCG1 does not alter synaptic plasticity

To test the capacity for synaptic plasticity in mice over-expressing ABCG1, we monitored the induction of long-term potentiation by applying high-frequency stimulation (four bursts of 50 pulses at 100 Hz, 30 s between bursts) to the Shaeffer collaterals of the CA1. Hippocampal slices from both wild-type and ABCG1 BAC Tg animals demonstrated robust long-term potentiation, suggesting intact synaptic plasticity in transgenic animals (Figure 3C). In addition to long-term potentiation, a paired-pulse protocol (50 inter-stimulus interval) was employed before and after the high-frequency stimulation tetanus as a measure of presynaptic properties [10]. Standard paired-pulse facilitation was observed in both wild-type and ABCG1 BAC Tg animals (Figure 3D). Taken together, these results indicate that the selective over-expression of ABCG1 does not affect synaptic plasticity in the CA1.

## Discussion

The present experiments investigated the effects of ABCG1 over-expression on both baseline behavioural phenotype and cognition. ABCG1 BAC Tg mice were indistinguishable from wild-type animals when assessed using the SHIRPA protocol, indicating that over-expression of ABCG1 does not result in any gross metabolic disturbances that could affect observable behavioural or physiological measures. Cognitive measures were also demonstrated to be unaffected in transgenic animals. The performance of ABCG1 BAC Tg mice on tasks measuring anxiety, general locomotor activity and spatial learning and memory were all comparable to those of wild-type animals. Additionally, our results show that stimulation of Schaeffer axon collaterals elicits comparable long-term potentiation in both ABCG1 BAC Tg and wild-type animals. No significant difference in paired-pulse facilitation was observed between ABCG1 BAC Tg mice and wild-type littermates both prior to, and after tetanus stimulation, suggesting that the presynaptic properties of ABCG1-over-expressing mice are intact. These data strongly suggest that over-expression of ABCG1 alone is not sufficient to cause changes in cognition, learning and memory or hippocampal synaptic plasticity.

While ABCG1 over-expression does not elicit a change in cognition, ABCG1 over-expression appears to alter the brain at the cellular level. Although total brain cholesterol levels are unaffected, over-expression of ABCG1 leads to decreased levels of sterol precursors and 24S-hydroxycholesterol in the brain, suggesting that ABCG1 suppresses flux through the cholesterol biosynthetic pathway [6]. There are several possible explanations for the lack of transgenic phenotype. It is possible that the changes caused by over-expression of ABCG1 may be too subtle to have globally obvious effects on cognition, producing changes at the neuronal level that may be below the detection threshold of the behavioural and cognitive tests used in our experiments. Another possible explanation for the lack of transgenic phenotype is the existence of a compensatory pathway that acts to balance out the effects of ABCG1 over-expression and maintain normal cognitive functions in the brain. The ABCG4 transporter could potentially act in this manner, as it is highly homologous to ABCG1 and is co-expressed with ABCG1 in neurons and astrocytes [11]. Moreover, ABCG4 has also been proposed to be important in cholesterol homeostasis; both ABCG1-deficient and ABCG4-deficient mice each exhibit repression of SREBP-2 target genes involved in cholesterol synthesis, suggesting that ABCG4 does function in regulation of cholesterol levels. It is possible that the ABCG4 pathway compensates for imbalances caused by over-expression of ABCG1, preventing major changes at the level of cognition. Further studies will be required to determine the exact functions of ABCG4 in brain cholesterol homeostasis, and whether it is indeed part of a pathway that could compensate for alterations in ABCG1 function.

## Conclusion

In conclusion, no effect of ABCG1 over-expression was seen in hippocampal synaptic plasticity and behavioural parameters such as anxiety and general locomotor activity, or on learning and memory, suggesting that despite changes in cholesterol flux in brain, it is unlikely that over-expression of ABCG1 contributes to cognitive deficits in the DS population.

## Competing interests

The authors declare that they have no competing interests.

## Authors' contributions

PP participated in planning the study, carried out behavioural and cognitive testing, performed statistical analysis of the data and assisted with manuscript preparation. TK carried out electrophysiology procedures, performed data analysis and drafted the manuscript. BE performed electrophysiological experiments and data analysis. BB performed perfusions and immunoblotting, and participated in the planning of the study. CW and BC contributed to planning of the experiments and discussion of results, and co-supervised the study.
